# Absolute quantification of cell-free microRNAs in cancer patients

**DOI:** 10.18632/oncotarget.3859

**Published:** 2015-05-02

**Authors:** Manuela Ferracin, Laura Lupini, Irene Salamon, Elena Saccenti, Maria Vittoria Zanzi, Andrea Rocchi, Lucia Da Ros, Barbara Zagatti, Gentian Musa, Cristian Bassi, Alessandra Mangolini, Giorgio Cavallesco, Antonio Frassoldati, Stefano Volpato, Paolo Carcoforo, Alan B. Hollingsworth, Massimo Negrini

**Affiliations:** ^1^ Department of Morphology, Surgery and Experimental Medicine, University of Ferrara, Ferrara, Italy; ^2^ Laboratory for Technologies of Advanced Therapies (LTTA), University of Ferrara, Ferrara, Italy; ^3^ Section of Hematology, S. Anna University Hospital, Ferrara, Italy; ^4^ Breast Unit, S. Anna University Hospital, Ferrara, Italy; ^5^ Section of General and Thoracic Surgery, S. Anna University Hospital, Ferrara, Italy; ^6^ Clinical Oncology Unit, S. Anna University Hospital, Ferrara, Italy; ^7^ Department of Medical Sciences, University of Ferrara, Ferrara, Italy; ^8^ Department of Surgery, Mercy Hospital – OKC, Oklahoma City, OK, USA

**Keywords:** cell-free microRNA, droplet digital PCR, breast cancer, cancer biomarkers

## Abstract

The hypothesis to use microRNAs (miRNAs) circulating in the blood as cancer biomarkers was formulated some years ago based on promising initial results. After some exciting discoveries, however, it became evident that the accurate quantification of cell-free miRNAs was more challenging than expected. Difficulties were linked to the strong impact that many, if not all, pre- and post- analytical variables have on the final results. In this study, we used currently available high-throughput technologies to identify miRNAs present in plasma and serum of patients with breast, colorectal, lung, thyroid and melanoma tumors, and healthy controls. Then, we assessed the absolute level of nine different miRNAs (miR-320a, miR-21-5p, miR-378a-3p, miR-181a-5p, miR-3156-5p, miR-2110, miR-125a-5p, miR-425-5p, miR-766-3p) in 207 samples from healthy controls and cancer patients using droplet digital PCR (ddPCR) technology. We identified miRNAs specifically modulated in one or more cancer types, according to tissue source. The significant reduction of miR-181a-5p levels in breast cancer patients serum was further validated using two independent cohorts, one from Italy (*n* = 70) and one from US (*n* = 90), with AUC 0.66 and 0.73 respectively. This study finally powers the use of cell-free miRNAs as cancer biomarkers and propose miR-181a-5p as a diagnostic breast cancer biomarker.

## INTRODUCTION

The detection of changing molecules in diseased people blood is the rationale behind many diagnostics procedures. This final goal can be reached only after a careful identification of suitable biomarkers, whose accuracy could significantly overcome the probability to make a casual diagnosis. The use of early-detection or residual-disease biomarkers is especially necessary in the case of cancer disease where this step may represent a major difference in clinical outcome.

Soon after their discovery as cell-free blood molecules, microRNAs (miRNAs) have been proposed as potential disease biomarkers because they are stable, retrievable and measurable from fresh or archival serum and plasma samples [1-3]. In addition, circulating miRNA levels were soon described as significantly different between healthy and diseased subjects with any major cancer type [4–9]. Despite the promising results, several concerns still exist. Firstly, the impact of every pre-analytical step is so strong that prevents the reproducibility of miRNA assessments in published studies [10]. Secondly, the source choice (mainly serum, plasma) and its processing has very relevant consequences on miRNA concentration [11]. Thirdly, data normalization has been performed in a variety of ways, often without providing adequate data supporting the choice of a specific “reference” gene or method, thereby generating plenty of non-comparable results. Indeed, many studies have been performed using RT-qPCR technology and selecting a circulating “reference” gene according to information derived from tissue samples (commonly small nucleolar RNAs or miR-16) without an accurate verification of their stability in the circulation. Moreover, miR-16 has been reported to be a circulating cancer biomarker itself in several studies [12-14] and the RNU6B instability in circulation has been recently demonstrated [15]. In this host of contradictory information, solid and reliable miRNA biomarkers run the risk of being blurred and ignored.

Recently, a comparison of miRNA quantification systems and platforms using body-fluids derived miRNAs has been performed, in the context of a more comprehensive study, by Mestdagh and colleagues [16]. This study highlighted the lower sensitivity of hybridization and sequencing technologies when quantifying serum miRNAs if compared to qPCR platforms. In addition, Hindson et al. [17] demonstrated that, for quantifying circulating miRNAs, the droplet digital PCR system (Bio-Rad Laboratories) was superior to conventional qPCR, allowing also an absolute quantification. Moreover, ddPCR proved to be more tolerant than qPCR to the presence of inhibitors in the amplification reaction [18].

Therefore, we decided to assess the absolute levels of the most abundant cell-free miRNAs in the serum and plasma of breast, colorectal, lung, melanoma patients using droplet digital PCR (ddPCR) technology and EvaGreen-based miRNA assays [19] to verify their utility as diagnostic biomarkers.

## RESULTS

### Qualitative analysis of cell-free miRNAs

We performed a qualitative screening of miRNAs species circulating in serum and plasma of breast, colorectal, lung, melanoma patients using either microarray or small RNA sequencing. The microRNA expression profile of 80 plasma samples was generated using Agilent miRNA microarrays. We hybridized fixed volumes of total RNA, derived from fixed plasma volumes, for 18 breast cancer (BC), 18 colorectal cancer (CRC), 18 lung cancer (LC), 8 melanoma (M) and 18 healthy control (C) samples. Bioinformatics analyses revealed that 255 miRNAs were expressed in at least one sample (Supplementary Table 1). Microarray experiments from plasma samples revealed a reduced consistency between samples from the same tumor type, if compared to other experiments performed with low-abundance RNA samples [20]. Indeed, we found a poor correlation between patients with the same tumor (Supplementary Figure 1). We used microarray data to obtain the global miRNA expression profile of all individual cancer types and control group.

We identified the most abundant cell-free miRNAs using also a small RNA-seq approach with Ion Torrent PGM platform (Life Technologies) for massive parallel sequencing. We sequenced 7 pools of 10 samples each (plasma: C, BC, CRC, LC, M and serum: C, BC). Although the number of reads per miRNA was quite low (average reads count = 4), we identified 215 miRNAs detected in at least 1 sample (Supplementary Table 2).

### Absolute quantification of candidate miRNA biomarkers using ddPCR

We used the results obtained from microarray and small RNA-seq analyses to identify a panel of miRNAs showing a potential different abundance in the plasma (microarray and NGS) or serum (NGS) of four cancer types (BC, LC, CRC, M) compared to healthy controls. We selected the miRNA candidates between the most counted in either microarray or NGS experiments and the potentially modulated, according to fold change analysis. We selected the following miRNAs: miR-320a, miR-21-5p, miR-378a-3p, miR-181a-5p, miR-3156-5p, miR-2110, miR-125a-5p, miR-425-5p, miR-766-3p. We assessed the absolute levels of these selected miRNAs using EvaGreen-based ddPCR technology [19] and constant volumes throughout the experiments [17, 19].

We established a two-step approach. In the first step we assessed the absolute miRNA levels using ddPCR technology in a pilot cohort comprising 20 subjects per group, with the exception of melanoma for which we had only 10 subjects available. We tested a selected miRNA (miR-181a-5p) also in a group of 27 thyroid cancers. We assessed the miRNAs both in plasma (miR-320a, miR-21-5p, miR-378a-3p, miR-181a-5p, miR-3156-5p, miR-2110, miR-125a-5p, miR-766-3p) and serum (miR-320a, miR-21-5p, miR-378a-3p, miR-181a-5p, miR-125a-5p, miR-425-5p). Then, we used the information obtained from miRNA data distribution to determine the samples size necessary to confirm a promising preliminary result and, as a second step, we extended the assessment to an adequate number of subjects in order to obtain the desired statistical power.

**Figure 1:**
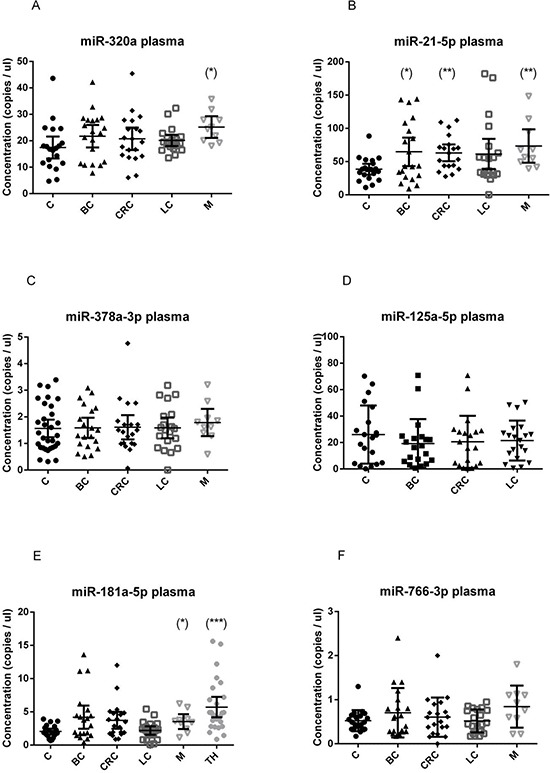
Quantification of miRNA levels in plasma Concentration of miR-320a **A.** miR-21-5p **B.** miR-378a-3p **C.** miR-125a-5p **D.** miR-181a-5p **E.** miR-766-3p **F.** detected using EvaGreen ddPCR, in the plasma of 20 breast cancer (BC), 20 colorectal cancer (CRC), 20 lung cancer (LC), 10 melanoma (M) and 20 healthy control (C) samples. miR-181a-5p was quantified also in 27 thyroid cancer samples (TH). Results are presented as copies per microliter of the amplification reaction mixture. The difference between cancer samples and controls was evaluated for significance using the two-tailed, unpaired *t*-test (*p < 0.05; **p < 0.005; ***p < 0.0005).

**Figure 2:**
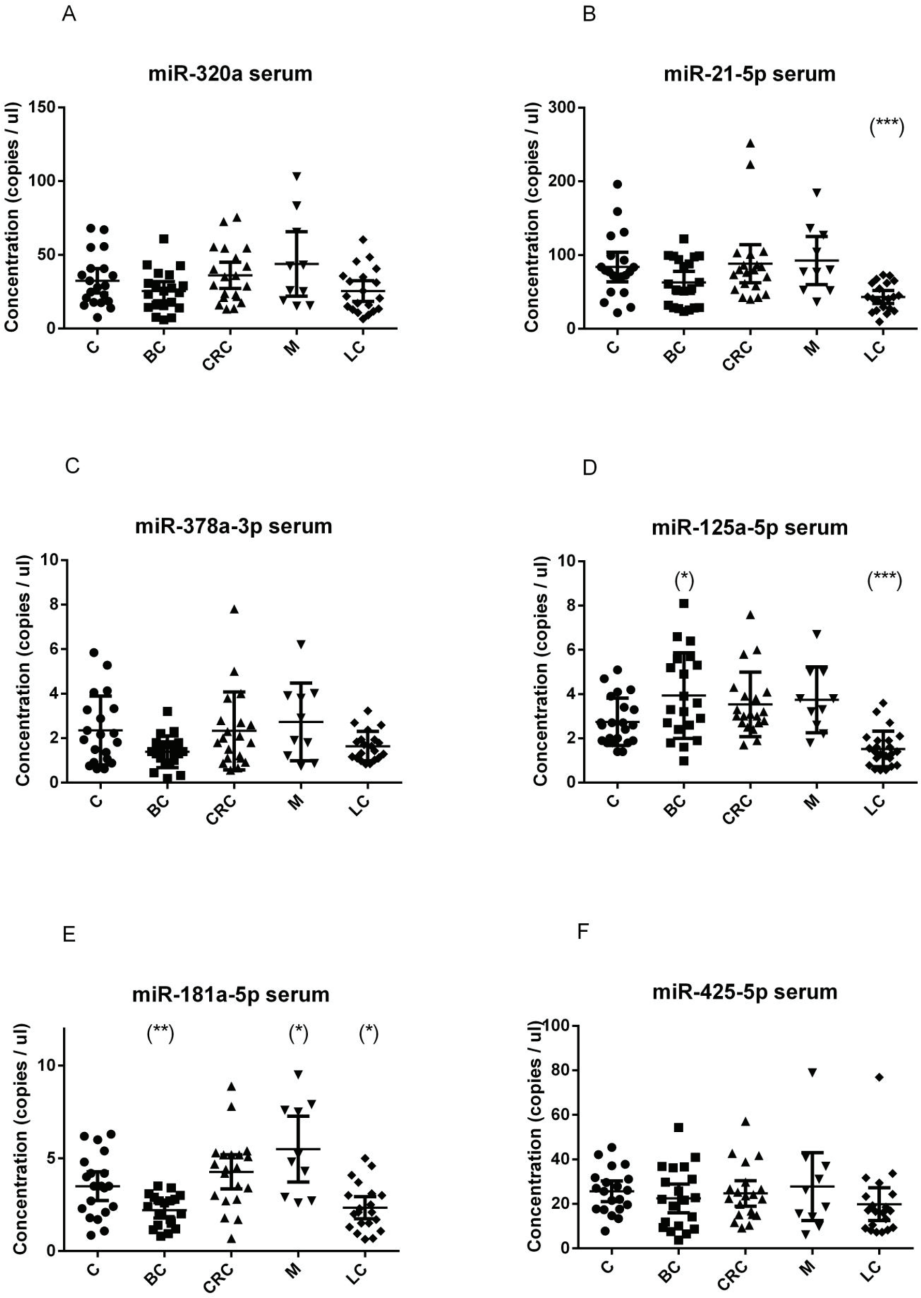
Quantification of miRNA levels in serum Concentration of miR-320a **A.** miR-21-5p **B.** miR-378a-3p **C.** miR-125a-5p **D.** miR-181a-5p **E.** miR-425-5p **F.** detected using EvaGreen ddPCR, in the serum of 20 breast cancer (BC), 20 colorectal cancer (CRC), 20 lung cancer (LC), 10 melanoma (M) and 20 healthy control (C) samples. Results are presented as copies per microliter of the amplification reaction mixture. The difference between cancer samples and controls was evaluated for significance using the two-tailed, unpaired *t*-test (*p < 0.05; **p < 0.005; ***p < 0.0005).

We found that the investigated miRNAs exhibited similar levels and data distribution across all cancer types (Figure 1, A–F and Figure 2, A–F).

In plasma samples, we observed a significant up-regulation of miR-320a in subjects with melanoma (*p* = 0.0077) (Figure 1A), of miR-181a-5p in melanoma (*p* = 0.03) and thyroid cancer patients (*p* < 0.0001; AUC 0.87) (Figure 1E), of miR-21-5p in breast (*p* = 0.024), melanoma (*p* = 0.012) and colorectal cancer patients (*p* = 0.0013) (Figure 1B), if compared to healthy subjects. In plasma, miR-3156-5p and miR-2110 were not detectable (data not shown) and were not tested further.

In serum samples, miR-21-5p and miR-125a-5p were significantly down-regulated in lung cancer patients (*p* < 0.001) (Figure 2B, 2C); miR-181a-5p was up-regulated in melanoma (*p* = 0.013) and down-regulated in the serum breast cancer patients (*p* = 0.004) (Figure 2F), and was further explored in additional samples (see a following section).

The absolute amount of each miRNA species was found to be very different, being miR-21-5p the most abundant, both in serum and in plasma, followed by miR-320a. Assuming that each miRNA molecule is reverse transcribed in a cDNA molecule, we can calculate the absolute copies in 1 μl of plasma or serum multiplying the obtained concentration in ddPCR reaction value for a dilution factor (145.83).

### Differences in miRNA levels according to the source

**Figure 3:**
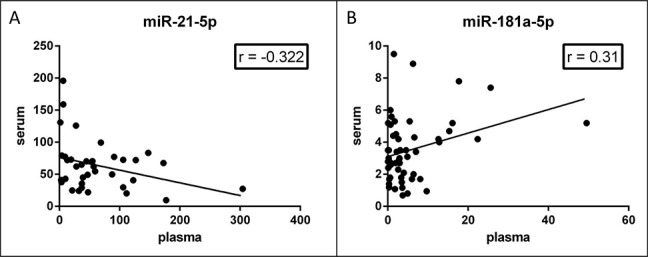
Correlation of miRNA levels in matched plasma-serum Concentration of miR-21-5p **A.** and miR-181a-5p **B.** in paired serum and plasma from the same subject. miR-21-5p levels are inversely correlated (Pearson *r* = −0.32, *p* = 0.055); miR-181a-5p levels are positively correlated (Pearson *r* = 0.31, *p* = 0.023) in the two tissues.

We assessed miR-21-5p (Figure 3A) and miR-181a-5p (Figure 3B) levels in randomly selected, matched, plasma and sera from the same patient. Considering that plasma was prepared using a one-step centrifugation at 1000 x *g*, we found substantially different levels of the same miRNA in the two tissues. Pearson correlation index was −0.32 (*p* = 0.055) for miR-21-5p and 0.3 (*p* = 0.024) for miR-181a-5p. These results suggested that the correlation of miRNA levels in matched plasma and serum can vary for each miRNA species and rely strongly on tissue preparation.

### Absolute levels of miR-181a-5p are reduced in breast cancer serum in two independent cohorts

**Figure 4:**
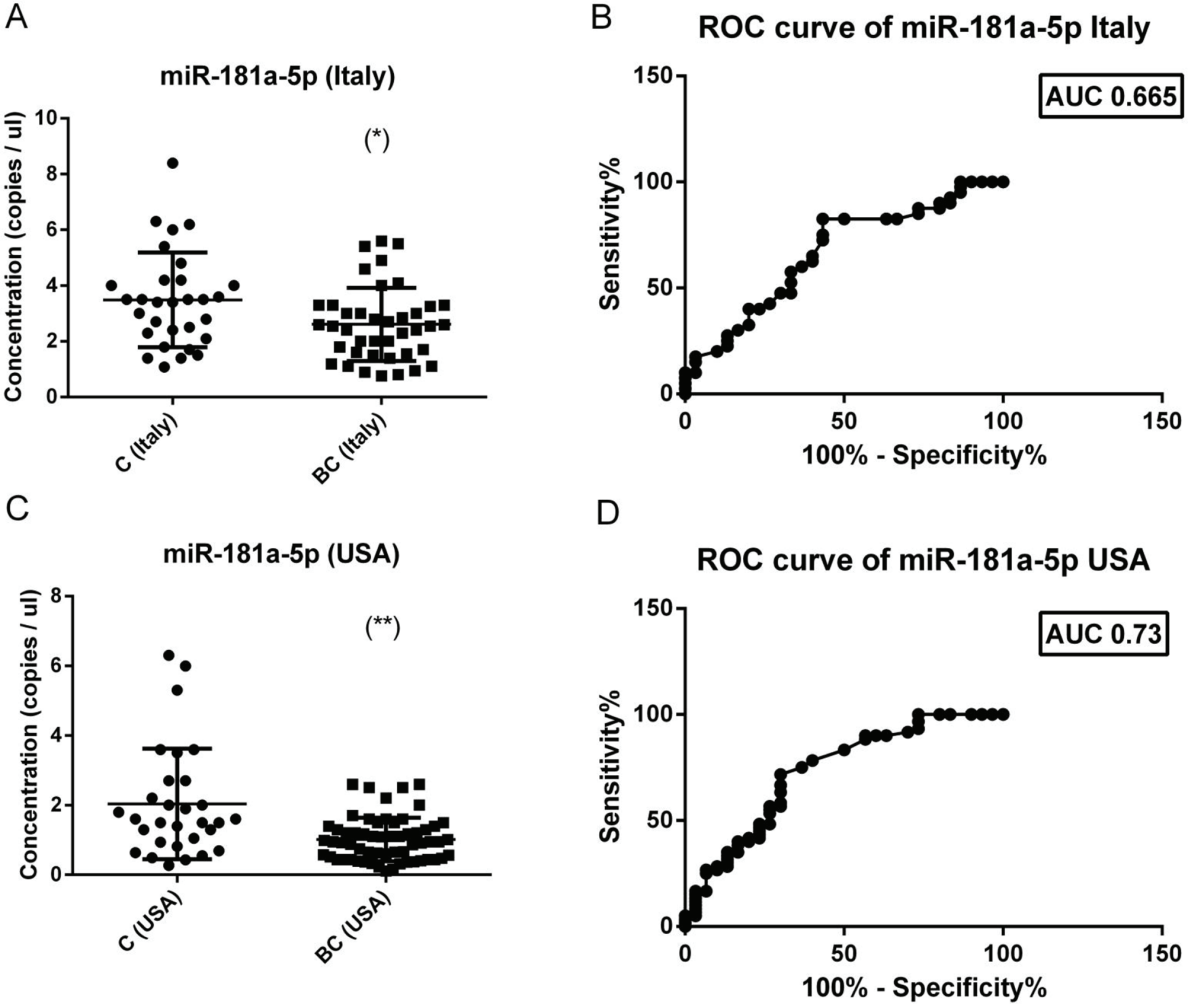
Absolute levels and ROC curves of miR-181a-5p in two different cohorts of breast cancers The concentration (copies / μl) of miR-181a-5p was calculated in two independent cohorts (Italy and USA) of breast cancers (40 Italy + 60 USA) and healthy controls (30 Italy + 30 USA. In both cohorts there is a significant reduction of miR-181a-5p levels in breast cancer sera (left panel). ROC curve analysis of miR-181a-5p levels revealed an AUC of 0.665 for the italian cohort (right upper panel) and of 0.73 for the american cohort (right lower panel). The difference between breast cancer samples and controls was evaluated for significance using the two-tailed, unpaired *t*-test (*p < 0.05; **p < 0.005).

We validated the miR-181-5p down-regulation in a larger group of breast cancer patients. We selected miR-181-5p because we had two independent cohorts available, one collected in Ferrara (Italy cohort, 2010-2014) and one collected in Oklahoma (USA cohort, 2005-2013). Patients clinical-pathological are reported in Table 1 and Supplementary Table 3. We found a significant down-regulation of miR-181a-5p in both cohorts of breast cancer patients compared to their matched controls (Figure 4). The diagnostic performance of miR-181a-5p was assessed by constructing a receiver operating characteristic (ROC) curve and calculating the area under each ROC curve (AUC). For the discrimination of patients with breast cancer from healthy individuals, the AUC for the Italy cohort was 0.665 (*p* = 0.01) (95% confidence interval, 0.535–0.795) and the AUC for the Oklahoma cohort was 0.73 (*p* < 0.001) (95% confidence interval, 0.614–0.845). These results suggested that miR-181a-5p is a potential biomarker for breast cancer diagnosis.

**Table 1:** Clinico-pathological features of breast cancer patients used in serum analyses

Characteristics		Breast Cancer Italy (*n* = 40)	Breast Cancer USA (*n* = 60)
**Hystological subtype**	Ductal	30 (75%)	49 (81%)
	Lobular	6 (15%)	4 (7%)
	Tubular	1 (2.5%)	1 (2%)
	other	3 (7.5%)	6 (10%)
**Lymph node involvement (pN)**	pN0	30 (75%)	32 (54%)
	pN1	8 (20%)	18 (30%)
	pN2	0 (0%)	7 (12%)
	pN3	1 (2.5%)	1 (2%)
	pNx	1 (2.5%)	1 (2%)
**Stage**	0	0 (0%)	3 (5%)
	I	23 (58.5%)	17 (28%)
	II	15 (38.5%)	28 (47%)
	III-IV	1 (2.5%)	12 (20%)
	unknown	1 (2.5%)	
**Grade**	I	4 (10%)	8 (14%)
	II	27 (67.5%)	12 (20%)
	III	9 (22.5%)	40 (66%)
**Estrogen receptor**	positive	38 (95%)	41 (68%)
	negative	2 (5%)	17 (28%)
	unknown	0 (0%)	2 (4%)
**Progesterone receptor**	positive	34 (85%)	34 (56%)
	negative	6 (15%)	24 (40%)
	unknown	0 (0%)	2 (4%)
**HER2/neu receptor**	positive	9 (22.5%)	12 (20%)
	negative	31(77.5%)	42 (70%)
	unknown	0 (0%)	6 (10%)

We evaluated the association of miR-181a-5p levels with the following clinical-pathological parameters: histological subtype, stage, grade, nodal status, molecular subtype, ER/PR status, HER2 status, proliferation index. There was no significant association between miR-181a-5p levels and these parameters.

## DISCUSSION

In our study, we assessed the absolute levels of miRNA molecules circulating in plasma and serum body fluids obtained from subjects with four main different cancer types. Quantification of cell-free miRNAs has recently come to the fore as a potential source of cancer disease biomarkers. Anyway, miRNA quantification has been performed in a variety of manners, thus generating poorly overlapping and hardly reproducible results [21].

Here, we performed a screening to identify the most abundant miRNA species in plasma and in serum using both microarray and small-RNA sequencing technologies. We verified that currently available high-throughput technologies display poor performance in circulating miRNA quantification, as recently confirmed also by the miRQC study [16]. Therefore we used these technologies only to identify a panel of high-abundance, potentially deregulated miRNAs. As far as we could see, the cell-free global miRNA profile does not correspond to that of solid tumors and the most differently abundant miRNAs do not reflect the changes in tumors, with one exception that is miR-21. Assuming that cell-free miRNAs could be released by neoplastic cells, researchers have frequently tried to validate as circulating cancer biomarkers the same miRNAs that were de-regulated in solid tumors. This assumption is not confirmed by our observed data, and it is in line with results reported by Jarry et al. [10] and by Cookson et al. [22].

In our validation step, we used the droplet digital PCR technology to get an accurate absolute quantification of specific cell-free miRNAs, working in every step with constant volumes. Digital PCR can be considered superior to RT-qPCR in circulating miRNA quantification, because of its higher sensitivity and reduced variability in low-abundance miRNA detection [17]. By this way, we have been able to perform an absolute quantification of miRNA copies, without the need of additional normalization steps. Therefore, results obtained using droplet digital PCR can be easily used to calculate the number of each miRNA molecule that are present in one ml of plasma or serum.

Another issue that is frequently underestimated in miRNA biomarkers studies concerns with the plasma and serum preparation. In our experience, every change in tissue collection steps (like source type, blood tube used, tissue centrifugation and conservation) determines changes in miRNA levels, as confirmed by published studies [23, 24]. Thereby we cannot compare the miRNA profiles across studies in which tissues have been collected using different protocols. Moreover, in several studies this information is missing in the published methods. The RNA extraction procedures and the quantification assay have a relevant impact as well. In our study, we give a detailed description of all pre-analytical steps thus guarantying the possibility to reproduce the presented results. According to our sample processing (especially plasma centrifugation rate), the absolute levels of the same miRNA in sera were different than those in plasma, and their difference depend on the miRNA species under investigation (as reported in Figure 3). This information is relevant to avoid biased results when performing meta-analyses of miRNA biomarkers data. We hypothesized that the main differences observed between serum and plasma could be linked to the permanence of microvescicles and exosomes, that contains microRNAs [25], in our plasma preparations. Indeed, we know that stronger centrifugation steps (3200xg minimum) are necessary to remove most platelets [11] and some part of microvescicles. Here, we decided to use a microvescicle-rich plasma to gain also microvescicles and exosomes miRNA contribution. According to this hypothesis, circulating miRNAs linked to Argonaute protein or HDL will display similar levels in plasma and serum, while exosome and microvescicle encapsulated miRNAs will display different levels, since microvescicles are trapped inside serum clot and discharged.

Despite all the technical issues, our data clearly indicate that miRNA molecules are present in the circulation at levels that change according to the cancer status. They can be assessed, although with different outcomes, both in serum and plasma. We quantified eight different miRNAs in plasma and six miRNAs in sera obtained from patients with four different cancer types (five in the case of plasmatic miR-181a-5p) and healthy controls. We found only one miRNA, miR-21-5p, consistently increased in the plasma of all cancer patients. In plasma, other miRNAs were more cancer specific like miR-320a for melanoma and miR-181a-5p for melanoma and thyroid cancer.

In serum, three miRNAs displayed reduced levels in lung cancer (miR-21-5p, miR-125a-5p and miR-181a-5p). According to plasma results, miR-181a-5p was more abundant in the serum of melanoma patients than in controls. In our pilot cohort of twenty subjects per cancer type, miR-181a-5p was reduced in the serum of breast cancer patients. To validate this observation, miR-181a-5p, as a potentially biomarker miRNA for breast cancer, was further assessed in the serum of two extended cohorts of breast cancer patients, collected in different countries (USA and Italy) and in different years. In published studies, circulating miR-181a (now miR-181a-5p) levels have been described as reduced in the blood of Luminal A breast cancer [26]; McDermott and colleagues assessed miR-181a in a cohort of 44 patients and 46 controls by RT-qPCR starting from whole blood RNA, using miR-16 for normalization. Reduced miR-181a levels in breast cancer were documented also by Guo et al [27] using a custom made stem-loop PCR to quantify miR-18a/miR-16 levels in 152 breast cancer patients and 75 controls. Our results demonstrated that cell-free miR-181a-5p absolute levels are significantly lower in the serum of breast cancer patients, independently from the Institute where they were collected, if compared to time- and processing-matched healthy controls. We think that this information is very relevant for laboratories that plan to use circulating miRNA levels as diagnostic tools. Indeed, the reference range of miRNA levels that characterize the healthy state should be determined using an healthy population perfectly matched with the subjects demanding a diagnostic procedure.

Overall, this study establishes the basis for the use of miRNA absolute quantification with the digital PCR technology in miRNA biomarkers studies and confirms, in two independent populations, the potential of cell-free miRNAs, and specifically of miR-181a-5p, as minimally invasive breast cancer biomarkers.

## METHODS

### Ethics statement and blood samples

The study protocol was approved by the Ethics Committee of Ferrara University Hospital and of the Institutional Review Board of Mercy Hospital – OKC (USA). All participants provided written informed consent for the use of their samples for research purposes. All samples were collected prior to surgery and any therapy. At Ferrara University Hospital (Ferrara, Italy) peripheral venous blood was collected from 147 persons with breast, colorectal, lung, thyroid and melanoma tumors, and from age-matched 60 healthy donors (Supplementary Table 3). At Mercy Hospital (OK, USA), blood was collected from 90 patients, 60 of whom had their sample drawn pre-biopsy for what proved to be primary breast carcinoma. The 30 controls were drawn from subjects in a high-risk surveillance program, where women were followed with a combination of mammography and breast MRI. Subjects were designated as controls only when breast imaging was completely negative.

For plasma: 5 mL blood was collected in EDTA (ethylenediaminetetracetic acid) tubes (Vacuette); samples were centrifuged at 1000xg for 10 min to remove blood cells, and the supernatant plasma was dispensed in aliquots. For serum: at Ferrara Hospital and at Mercy Hospital, 5 mL blood was collected in red stopper clot tubes (BD Vacutainer). Tubes were kept at room temperature to clot for at least 60 min, and then spun at 1000xg for 10 min at room temperature; the serum was removed and dispensed in aliquots. Aliquots were stored at −80°C until use. Frozen samples were shipped from Oklahoma City (USA) to University of Ferrara on dry ice, and all samples remained frozen upon arrival.

Free haemoglobin concentration was analyzed by spectrophotometric analysis as described [28]. Hemolyzed samples were excluded from the analyses.

### RNA isolation

Total RNA including miRNA was extracted from 200 μl plasma or serum using the miRNeasy Mini Kit (cat. no. 217004, Qiagen) according to the manufacturer's supplementary protocol [29] with two minor variations. Specifically, after the sample was mixed with 1 ml QIAzol Lysis Reagent, 3 μl of a 4.16 nM solution of the synthetic miRNA cel-miR-39-3p from C. elegans (custom synthesized by Integrated DNA Technologies) was added. Also, RNA was eluted from spin columns in 35 μl nuclease-free water.

### Reverse transcription and ddPCR

For EvaGreen assays, 3 μl RNA was reverse-transcribed in a 20 μl reaction using the Universal cDNA synthesis kit II (Exiqon) following the company's guidelines for miRNA profiling in serum and plasma. PCR was performed in a 20 μl volume containing 10 μl 2X EvaGreen supermix (Bio-Rad), 8 μl diluted cDNA, and one of the following miRCURY LNA PCR primer sets (Exiqon): hsa-miR-320a (ID 204154), hsa-miR-21-5p (ID 204230), hsa-miR-378a-3p (ID 204179), hsa-miR-181a-5p (ID 204566), hsa-miR-3156-5p (ID 206999), hsa-miR-2110 (ID 204328), hsa-miR-125a-5p (ID 204339), hsa-miR-425-5p (ID 204337), hsa-miR-766-3p (ID 204499), cel-miR-39-3p (ID 203952). LNA primers were used as follow: 1 μl at 58°C for miR-320a, 0.25 μl at 56°C for miR-21-5p, 0.5 μl at 58°C for miR-378a-3p, 0.5 μl at 60°C for miR-181a-5p, 1 μl at 58°C for miR-3156-5p, 0.5 μl at 58°C for miR-2110, 0.5 μl at 58°C for miR-125a-5p, 1 μl at 56°C for miR-425-5p, 0.5 μl at 60°C for miR-766-3p, 0.5 μl at 58°C for cel-miR-39.

### Droplet digital PCR workflow

Droplet digital PCR was performed as described [19]. Briefly, each ddPCR assay mixture (20 μl) was loaded into a disposable droplet generator cartridge (Bio-Rad). Then, 70 μl of droplet generation oil for probes (Bio-Rad) was loaded into each of the eight oil wells. The cartridge was then placed inside the QX200 droplet generator (Bio-Rad). When droplet generation was completed, the droplets were transferred to a 96-well PCR plate (Eppendorf) using a Rainin multichannel pipet. The plate was heat-sealed with foil and placed in a conventional thermal cycler. Thermal cycling conditions were: 95°C for 5 min, then 40 cycles of 95°C for 30 s and 58°C for 1 min (ramping rate reduced to 2%), and three final steps at 4°C for 5 min, 90°C for 5 min and a 4°C indefinite hold. A no template control (NTC) and a negative control for each reverse transcription reaction (RT-neg) were included in every assay. Cel-miR-39 assay was performed to monitor RT reaction efficacy.

### MicroRNA microarray

We evaluated the plasma samples global miRNA expression profile using the Agilent miRNA microarrays (G4870A). This array is capable to assess the expression of 1200 human miRNAs. We evaluated 80 samples from four different cancer types and healthy controls. We used fixed volumes (15 μl) of RNA for the hybridization procedures. Experiments were performed as previously described [20]. Microarray raw data were imported and analyzed using GeneSpring GX 12 software (Agilent Technologies). Since a signal was detectable for at least 150 probes for each sample, we apply a quantile normalization before statistical analyses. Global correlation coefficients were calculated using GeneSpring software.

### Small-RNA sequencing

We prepared seven RNA pools by mixing 5 μl RNA from ten different samples each. RNA was concentrated to 3 μl using a SpeedVac concentrator (Savant). Small RNA libraries were prepared using Ion Total RNA-Seq Kit v2 (Life Technologies). Ion One Touch System and Ion One Touch v.2 DL kit (Life Technologies) were used to clonally amplify library fragments, starting from 210 × 10^6^ library molecules, according to Life Technologies protocols. Enriched template Ion Sphere Particles were loaded into one Ion 314 Chip and sequenced using Ion Torrent PGM. The total number of reads overlapping miRNA regions were identified using TMAP alignment software against hg19 and the Feature Counter plugin available in the Ion Torrent Suite.

### Statistical analysis

Statistical analyses were performed with GraphPad Prism 6 software (GraphPad software, CA, USA). Unpaired *t*-test was used for two-groups comparisons, Welch's correction was added when the variances were significantly different between the two groups. Two-sided *p*-value was always calculated. Two outliers were excluded in miRNA analysis of plasma samples (lung cancer group) after running GraphPad outlier analysis (ROUT method). Correlation analysis between individual miRNAs in plasma/serum samples, ROC curve and AUC estimation were performed using GraphPad 6.0 software.

## SUPPLEMENTARY FIGURE AND TABLES









## Abbreviations

ddPCR: droplet digital PCR
miRNA: microRNA
MRI: magnetic resonance imaging
RT-qPCR: reverse transcription - quantitative polymerase chain reaction
ROC: Receiver Operating Characteristic
AUC: Area Under the Curve
