# Supplementary material for: Absolute quantification of cell-free microRNAs in cancer patients
Source: Oncotarget. 2015 May 2;6(16):14545–55. doi: 10.18632/oncotarget.3859 (PMC4546486; doi:10.18632/oncotarget.3859)
Supplement: Supplementary file 1 [file oncotarget-06-14545-s001.pdf]

SUPPLEMENTARY FIGURE AND TABLES

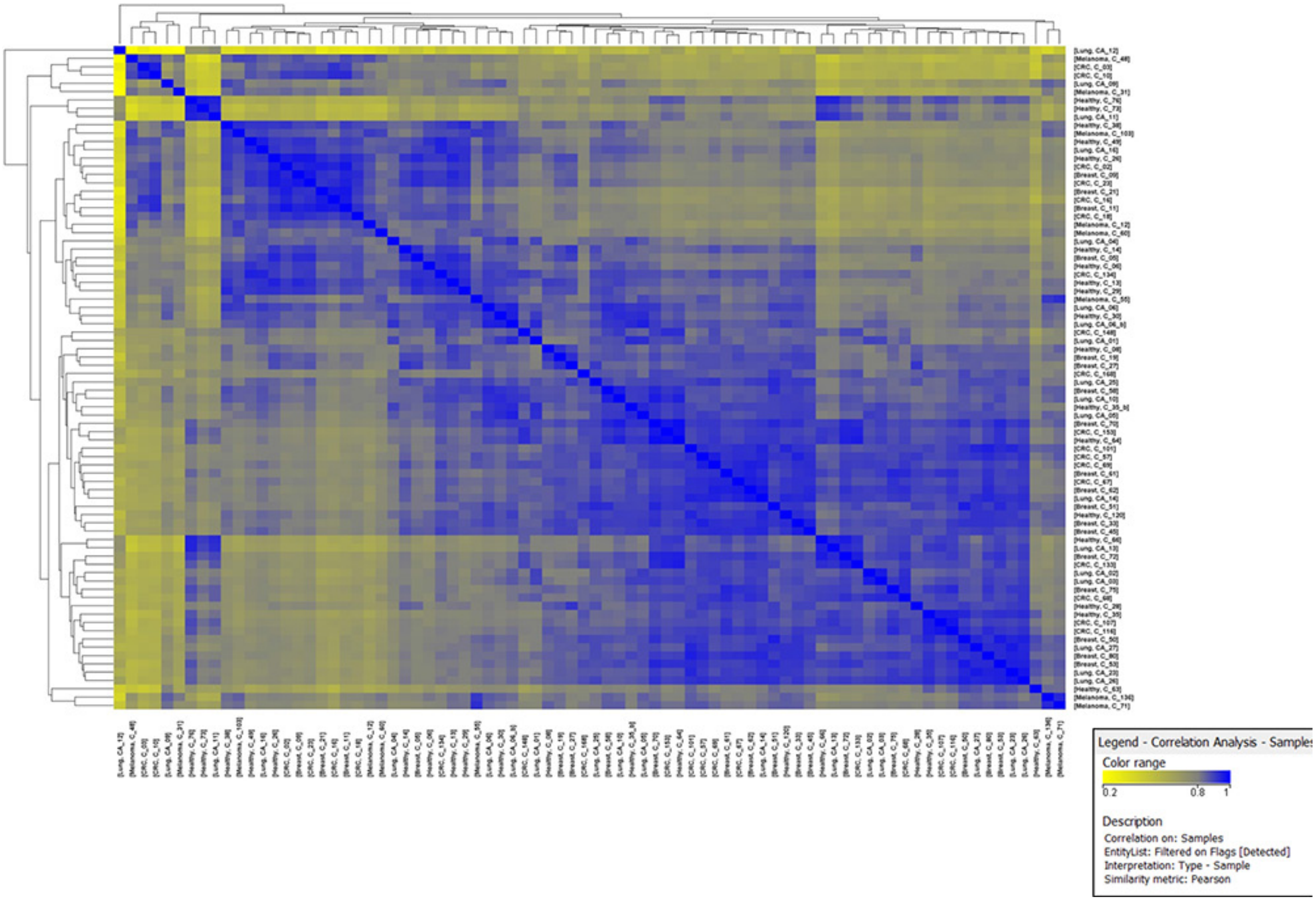

Supplementary Figure 1: Heatmap representation and cluster analysis of correlation values (Pearson index) obtained comparing the global miRNA expression profile of 80 samples hybridized on Agilent miRNA microarrays. Samples belonging to the same cancer type do not display a trend toward clustering in the same group, as expected.

Supplementary Table 1: Normalized, log2-transformed levels of 255 miRNAs detected in the plasma of cancer patients and healthy subjects by microarray technology

Supplementary Table 2: Levels of cell-free microRNAs measured by small-RNA sequencing. Reads Per Kilobase per Million (RPKM) values in plasma or serum pools of samples are reported

Supplementary Table 3: Clinical features of patients used in serum/plasma analyses
